# Substrate Stiffness and Stretch Regulate Profibrotic Mechanosignaling in Pulmonary Arterial Adventitial Fibroblasts

**DOI:** 10.3390/cells10051000

**Published:** 2021-04-23

**Authors:** Ariel Wang, Shulin Cao, Jennifer C. Stowe, Daniela Valdez-Jasso

**Affiliations:** Department of Bioengineering, University of California San Diego, La Jolla, CA 92093-0412, USA; arw020@ucsd.edu (A.W.); shc131@ucsd.edu (S.C.); jstowe@ucsd.edu (J.C.S.)

**Keywords:** pulmonary arterial hypertension, fibroblasts, computational modeling, stretch, stiffness

## Abstract

Pulmonary arterial adventitial fibroblasts (PAAFs) are important regulators of fibrotic vascular remodeling during the progression of pulmonary arterial hypertension (PAH), a disease that currently has no effective anti-fibrotic treatments. We conducted in-vitro experiments in PAAFs cultured on hydrogels attached to custom-made equibiaxial stretchers at 10% stretch and substrate stiffnesses representing the mechanical conditions of mild and severe stages of PAH. The expression of collagens α(1)I and α(1)III and elastin messenger RNAs (*Col1a1*, *Col3a1*, *Eln*) were upregulated by increased stretch and substrate stiffness, while lysyl oxidase-like 1 and α-smooth muscle actin messenger RNAs (*Loxl1*, *Acta2*) were only significantly upregulated when the cells were grown on matrices with an elevated stiffness representative of mild PAH but not on a stiffness representative of severe PAH. Fibronectin messenger RNA (*Fn1*) levels were significantly induced by increased substrate stiffness and transiently upregulated by stretch at 4 h, but was not significantly altered by stretch at 24 h. We modified our published computational network model of the signaling pathways that regulate profibrotic gene expression in PAAFs to allow for differential regulation of mechanically-sensitive nodes by stretch and stiffness. When the model was modified so that stiffness activated integrin β_3_, the Macrophage Stimulating 1 or 2 (MST1\2) kinases, angiotensin II (Ang II), transforming growth factor-β (TGF-β), and syndecan-4, and stretch-regulated integrin β_3_, MST1\2, Ang II, and the transient receptor potential (TRP) channel, the model correctly predicted the upregulation of all six genes by increased stiffness and the observed responses to stretch in five out of six genes, although it could not replicate the non-monotonic effects of stiffness on *Loxl1* and *Acta2* expression. Blocking Ang II Receptor Type 1 (AT_1_R) with losartan in-vitro uncovered an interaction between the effects of stretch and stiffness and angiotensin-independent activation of *Fn1* expression by stretch in PAAFs grown on 3-kPa matrices. This novel combination of in-vitro and in-silico models of PAAF profibrotic cell signaling in response to altered mechanical conditions may help identify regulators of vascular adventitial remodeling due to changes in stretch and matrix stiffness that occur during the progression of PAH in-vivo.

## 1. Introduction

Pulmonary arterial adventitial fibroblasts (PAAFs) are important for vascular extracellular matrix (ECM) homeostasis and remodeling [1,2], and there is evidence that PAAFs are regulated by matrix stiffness [3,4,5], stretch [6,7], or overstretch injury [8,9] and hypoxia [1]. During injury, PAAFs are activated and differentiate into myofibroblast subtypes that remodel vascular wall properties by directly altering the expression, degradation, or cross-linking of ECM proteins including collagen, fibronectin, and elastin. Given that the ECM also serves as a substrate for cell adhesion and sends physical and chemical cues that determine cell phenotype [10], it has been suggested that matrix stiffening may signal tissue remodeling and be causative drivers of pulmonary hypertension [11].

Pulmonary arterial hypertension (PAH) is a vasculopathy manifested by sustained elevation of pulmonary arterial pressures, vascular constriction, and irreversible vascular remodeling [1]. PAAFs in the adventitia of the pulmonary arterial wall are responsive to altered mechanical conditions and function to remodel the ECM, thereby modulating its mechanical properties. While fibroblast activation induces changes in the composition and structure of the vascular collagen matrix, it is unclear how PAAFs are regulated by matrix composition and stiffness, how PAAFs are affected by altered vessel stretch due to increased loading during PAH, what signaling pathways regulate these phenotypic responses to physical stimuli, and the extent to which these mechanically-stimulated pathways overlap and interact.

To better identify the receptors and pathways involved in regulating PAAF responses during PAH, we constructed a mechanosignaling model of PAAFs [12]. The computational model incorporated signaling pathways reported in the literature and a single input representing all types of mechanical stimulation [12]. Here we used an elaboration of this model together with in-vitro experiments on PAAFs cultured on different stiffness gels and under different stretch conditions to determine how six profibrotic genes respond to stretch and stiffness changes that mimic the mild and severe stages of PAH. The analysis suggests pathways that are differentially activated by changes in cell stretch and ECM stiffness that may help elucidate the sequence of tissue remodeling in PAAFs.

## 2. Materials and Methods

### 2.1. Cell Isolation

Pulmonary arteries (PAs) were harvested and isolated from six- to eight-week-old normotensive male Sprague–Dawley rats (Charles River Laboratories, Wilmington, MA, USA) under advisement of the Animal Care and Use Committee at the University of California San Diego (Protocol #S17237). The adventitial layer was stripped off and segments were cut into pieces, enzymatically digested with 1 mg/mL Type 2 collagenase (#LS004176, Worthington, Lakewood, NJ, USA) in Dulbecco’s Modified Eagle Media (DMEM, D5030, Gibco Thermo Fisher Scientific, Waltham, MA, USA) and agitated for 1.5 h at 37 °C, following the protocol by Liu et al. [13]. Fibroblast media was prepared by combining DMEM and 10% (v/v) fetal bovine serum (FBS) (#16140, Sigma Aldrich, St. Louis, MO, USA) and 1% antibiotic-antimycotic solution (#15240062, Gibco Thermo Fisher Scientific, Waltham, MA, USA). Isolated PAAFs were expanded on T75 tissue culture plastic (#25-209, Genesee Scientific, El Cajon, CA, USA) at 5% CO_2_, 37 °C, 100% humidity. To characterize PAAF cultures and compare their phenotypes to PAAFs in vivo, 10 mm segments of fixed, intact normotensive pulmonary artery were cryosectioned to 10µm and immunolabeled with antibodies against von Willebrand Factor (vWF) (#SC-365712, 1:50, Santa Cruz Biotechnology, Santa Cruz, CA, USA) as a marker for pulmonary arterial endothelial cells, myosin-11 (MYH11) (#SC-6956, 1:50, Santa Cruz Biotechnology, Santa Cruz, CA, USA) as a marker of pulmonary arterial smooth muscle cells, and vimentin (#AB-92547, 1:250, AbCam, Cambridge, UK) as a marker in all pulmonary artery cells that is also highly expressed in pulmonary arterial myofibroblasts, with appropriately matched secondary antibodies (Life Technologies, Carlsbad, CA, USA) (1:500) (Goat anti-Mouse Texas Red (#T862), Goat anti-Rabbit AlexaFluor700 (#A21038) with Wheat Germ Agglutinin-488 for membrane (#W6748, 10 μg/mL) and DAPI (#P36941) using standard immunofluorescence protocols with images taken at 40× magnification on a ThermoFisher Scientific EVOS FL-Auto 2 fluorescent microscope. The same staining protocol and imaging settings were also used to image isolated PAAFs that were expanded on plastic to characterize the culture. Cells were freshly isolated or used at a maximum passage number of 3 for these experiments. Data on Cytosoft^®^ 6-well plates (#5140 and #5142, Sigma Aldrich, St. Louis, MO, USA) were from seeding 100,000 frozen PAAFs per well, where 2 wells were pooled for RNA isolation after 3 days.

### 2.2. Stretcher Preparation

Polyacrylamide gels were prepared using stiffnesses corresponding to a normotensive pulmonary artery (0.5 kPa), mild PAH (3 kPa), and severe PAH (10 kPa), based on work by Liu et al. [14]. Gel stiffness was modulated by the percentage of acrylamide and bis-acrylamide (#A9099 and #146072, Sigma Aldrich, St. Louis, MO, USA): 3% acrylamide and 0.06% bis-acrylamide were used for the construction of 0.5 kPa gels; 4% acrylamide and 0.3% bis-acrylamide were used for the constructions of 3 kPa gels; and 10% acrylamide and 0.1% bis-acrylamide were used for the construction of 10 kPa gels [15].

Custom-made circular stretchers were designed using computer-aided design and constructed with polycarbonate. Polydimethylsiloxane (PDMS) membranes were built by mixing the Sylgard 186 elastomer kit (#4026144, Dow, Midland, MI, USA), extruding onto a wafer, degassing in a vacuum chamber then curing in the oven. The PDMS membranes were treated with 10% benzophenone (#A10739, Alfa Aesar Thermo Scientific, Haverhill, MA, USA) for polyacrylamide gel adherence, as previously described by Herum et al. [10]. The polyacrylamide gels were constructed to be 25 mm in diameter, cross-linked through exposure to ultraviolet light for 25 min, attached to PDMS membranes, and surrounded by silicone grease to prevent cell migration and media leakage. The gels were equilibrated in 1× Phosphate Buffered Saline (PBS, # 10010023, Gibco Thermo Fisher Scientific, Waltham, MA, USA) overnight, then Corning^®^ collagen I (100 g/mL, #354236, Sigma Aldrich, St. Louis, MO, USA) was attached with 1-Ethyl-3-(3-dimethylaminopropyl) carbodiimide (#00050, Chemplex, Mahwah, MJ, USA) and N-hydroxysuccinimide (#A10312, Alfa Aesar Thermo Scientific, Haverhill, MA, USA) to facilitate cell adherence. The stretcher was assembled so that two full turns were equivalent to 10% static stretch, as previously done [16,17]. PAAFs were trypsinized from the tissue culture plates using 0.25% Trypsin-EDTA (#25200056, Gibco Thermo Fisher Scientific, Waltham, MA, USA) and seeded onto the gels at a density of 140,000 cells per gel. Cells were cultured at 37 °C, 5% CO_2_, 100% humidity for three days. Cells were changed into serum-free media for 24 h before being stretched. The stretch condition was applied for 24 h based on the increase in gene expression shown by Herum et al. in left ventricular cardiac fibroblasts [10].

### 2.3. Inhibition Studies

For the inhibition experiments, PAAFs were seeded onto 0.5 kPa and 3 kPa gels at a density of 40,000 cells per gel and cultured for three days as described above. The media was changed to serum-free media, and each gel slated for inhibition was pre-incubated for 4 h with 1 M losartan (#3798, Tocris Bioscience, Minneapolis, MN, USA). The dose of losartan was delivered according to work by Kim et al. in adventitial fibroblasts from 6-week-old Sprague–Dawley rats [18]. The cells were then stretched for 24 h as previously described in the Stretcher Preparation subsection. RNA Isolation of these cells was conducted as described below.

### 2.4. RNA Isolation

For RNA extraction of the normotensive pulmonary artery, the adventitial layer was sectioned into 6 pieces and submerged in TRIzol reagent (Invitrogen #15596026, Thermo Fisher Scientific, Waltham, MA, USA). Tissue was homogenized using a BeadBug homogenizer with 1.5 mm zirconium beads. (Benchmark Scientific, Sayreville, NJ, USA). For PAAF experiments, RNA isolation was carried out using TRIzol and 5PRIME Phase Lock Gel Heavy tubes (#2302830, Quantabio, Beverly, MA, USA) and RNA was extracted using either a standard TRIzol total RNA isolation protocol or the RNeasy^®^ Mini kit (#74104, Qiagen^®^, Hilden, Germany), which was then reverse transcribed into cDNA using the NEB cDNA ProtoScript First Strand Kit (#E6300L, New England Biolabs, Ipswich, MA, USA) and then RNA was extracted using the RNeasy Mini kit (#74104, Qiagen, Hilden, Germany). Quantitative real-time PCR was performed using the StepOnePlus^TM^ Real-time PCR machine (Thermo Fisher Scientific, Waltham, MA, USA) and KAPA SYBR Fast Universal qPCR kit (#KK4601, Roche, Basel, Switzerland) using primers targeting genes of interest listed in Supplementary Table S1 (produced by Integrated DNA Technologies, San Diego, CA, USA). Relative gene expressions were compared against housekeeping gene 18S ribosomal RNA unless otherwise noted.

### 2.5. Imaging

Thirty thousand PAAFs were plated onto 35 mm cell culture dishes with #0 coverglass bottom (#D35-20-0-N, CellVis, Sunnyvale, CA, USA) onto 0.5 kPa or 3 kPa polyacrylamide gels, or directly onto plastic for 3 days at 37 °C and 5% CO_2_. Images were taken on an EVOS FL Auto 2 microscope, running software v2.0.1732.0 (Thermo Fisher Scientific, Waltham, MA, USA). Antibodies against Smooth Muscle alpha-Actin (mouse #A5228 1:100, Sigma, St. Louis, MO, USA) with secondary Goat anti-Mouse Texas Red (#T862, 1:250, Life Technologies, Carlsbad, CA, USA), Wheat Germ Agglutinin-488 for membrane (#W6748, 10 g/mL, Life Technologies, Carlsbad, CA, USA) and DAPI for nuclei in mounting media with Prolong Gold Antifade Reagent with DAPI (#P36941, Life Technologies, Carlsbad, CA, USA). Images were processed using DeconvolutionLab2 (EPFL, Lausanne, Switzerland) in ImageJ v1.53g4 developed by the National Institutes of Health (Bethesda, MD, USA).

### 2.6. Protein Quantification

Ten thousand PAAFs per gel were plated on 12 mm polyacrylamide gels at 0.5 kPa, 3 kPa, and 10 kPa stiffnesses formulated as described above and cultured for 3 days and fixed. Antibodies against Collagen 3a1 Rabbit (#13548-1-AP, 1:50, Proteintech, Wuhan, China) with secondary Goat anti-Rabbit AF700 (#A21038, 1:250, Life Technologies, Carlsbad, CA, USA) and against Smooth Muscle alpha-Actin (SMA) Mouse (#A5228, 1:100, Sigma, St. Louis, MO, USA) with secondary Goat anti-Mouse Texas Red (#T862, 1:250, Life Technologies, Carlsbad, CA, USA) were used to stain the PAAFs. The same imaging settings were used across cells cultured on different stiffnesses and fluorescence intensity was quantified using ImageJ v1.53g4 developed by the National Institutes of Health (Bethesda, MD, USA) and displayed as corrected total cell fluorescence (CTCF). Imaging data are added as Supplementary Table S2.

### 2.7. Computational PAAF Network Model

**Figure 1:**
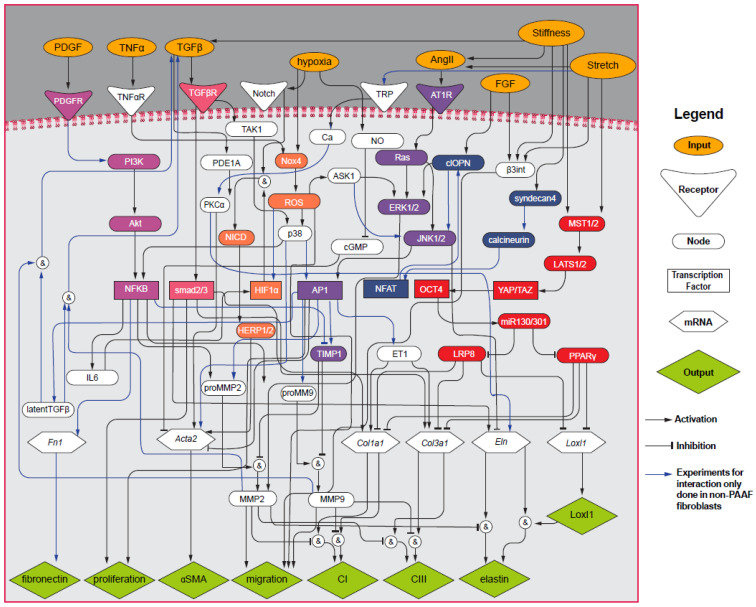
Pulmonary Arterial Adventitial Fibroblast (PAAF) mechanosignaling network with 8 input stimuli (orange ovals), 6 receptors (triangles), 34 nodes (ovals), 7 transcription factors (rectangles), 6 messenger RNAs (hexagons), and 8 phenotypic outputs (green diamonds), modified from our previous work [12]. Activation is shown with arrows and inhibition is shown with blunt head arrows. Blue arrows indicate non-PAAF-based experiments. Magenta nodes indicate the Phosphoinositide 3-kinase (PI3K) pathway, orange nodes indicate the Reactive Oxygen Species (ROS) pathway, purple nodes indicate the mitogen-activated protein kinase (MAPK) pathway, blue nodes indicate the calcineurin pathway, and red nodes indicate the Hippo signaling pathway.

We used our recently published PAAF network model [12] to investigate how substrate stiffness and stretch regulate profibrotic gene expression in PAAFs. The mechanical stimulus input was divided into substrate stiffness and stretch inputs, where stretch activated integrin β_3_, Ang II, MST1/2, and TRP; and stiffness activated integrin β_3_, Ang II, MST1/2, TGF-β, and Syndecan-4 [19,20,21,22,23,24,25]. We also added details to the activation of mitogen activated protein kinases (MAPKs) to allow independent regulation of JNK1/2, p38, and ERK1/2 [26,27,28,29,30,31,32,33,34]. In this refined model, ASK1 regulates JNK1/2 as well as ERK1/2 [26,27]. Ras was added downstream of AT_1_R to mediate regulation of ERK1/2 and JNK1/2 [28,29]. Based on studies by Xie et al. [30] in adult rat cardiac fibroblasts, the activation of JNK1/2 by cleaved osteopontin (clOPN) was included. The TGF-β receptor now also activates p38 via the TGF-β –activated kinase (TAK1) [31] and TGF-β receptor also activates *Eln* through smad2/3 based on work in PAAFs by Rabinovitch et al. [35]. Based on a model of cardiac fibroblasts by Zeigler et al. [32] and papers on MAPK signaling [33,34], we included ROS activation of p38. Finally, we incorporated the activation of TRP channels TRPC6 and TRPC1/C5 by stretch, which allows calcium to activate Protein Kinase C alpha (PKCα) [36,37,38] (Figure 1). Given the scarcity of PAAF studies, the network includes reactions from fibroblasts not derived from pulmonary arteries, such as cardiac and lung fibroblasts.

The PAAF signaling network model was implemented as a system of logic-based ordinary differential equations that were integrated numerically using an explicit Runge–Kutta method. Briefly, each state variable y_i_ that represents each of the species in the network is normalized to a value between 0 and 1 and follows a Hill-type activation function. The system of equations for the 69 nodes in the model takes the form:

(1) $$\frac{{\mathrm{dy}}_{i}}{\mathrm{dt}}=\frac{1}{\tau_{y_{i}}}\left(\omega_{i}y_{i}f\left({\mathrm{EC}}_{50},n,B\right)y_{i}^{\max}-{\;y}_{i}\right)$$

where ω_i_ represents the weight for each node, τ is the time constant, and f is the sigmoidal Hill function of y_i_, where f(EC_50_, n, B) is a function of half-maximal activation EC_50_, cooperativity n, and constant B. The form of biochemical interactions between two species is specified by ‘AND’ or ‘OR’ Boolean operators that represent the biochemical interactions between upstream nodes expressed as continuous functions as described in Wang et al. [12].

Inputs of the model (PDGF, TNF, TGF-β, hypoxia, AngII, FGF, stiffness, stretch) were initialized at 0.25. Default reaction weights ω_i_, Hill coefficient n, and half-maximal activation EC50 were set to 1, 1.4, and 0.6, respectively. The time constants τ for each different reaction followed those used previously [12]: 0.1 h for signaling reactions; 1 h for transcription; and 10 h for translation. Similar to the network model analysis by Tan et al., (2017), we chose a change in normalized model output values of 0.1 as the threshold for considering the output to have changed significantly by mechanical stimulation or for a significant response to have been significantly inhibited [39]. While Tan et al., (2017) used a threshold of 0.05, we chose a more stringent threshold of 0.1, but our conclusions were not affected by this difference. Parameters in the model were not optimized or fitted. Rather we chose equal parameters for all reactions using values from Zeigler et al. [32]. While the parameters EC_50_, weight, and Hill coefficient were set to be the same value across all reactions, the time constant τ was chosen according to the type of reaction. Furthermore, parameter values were tested for consistency with the mathematical constraints described in Cao et al. for this class of model [36]. In a previous comprehensive analysis of parameter uncertainty, we verified that model accuracy was robust to the choices of these parameter values [12].

The input weights of stretch and stiffness were both set to 0.25 to represent the softest matrix, 0.5 kPa, and no applied stretch. We increased the stiffness input weight to 0.7 and 0.9 to represent the effects of 3 kPa and 10 kPa substrates, respectively, and evaluated the model at t = 72 h to mimic the in-vitro experimental time course.

To numerically simulate the effects of stretch on PAAFs after 24 h and the changes in substrate stiffness for 72 h when inhibiting nodes, the model was evaluated at those time points (i.e., y_i_ (t = 24) and y_i_ (t = 72)) after the corresponding input weights of stiffness and stretch were increased from 0.25 to 0.7. To simulate the effects of inhibition, y_i_^max^ corresponding to blocked nodes were set to 0, while the other parameters remained the same. The change for each gene was calculated with respect to each condition’s control group.

To simulate the different conditions under which losartan inhibited AT_1_R, we conducted eight sets of simulations. Four sets of these simulations were evaluated on 0.5 kPa substrate stiffness for 72 h with parameters at baseline, and input weights stiffness and stretch set to 0.25. For simulations involving stretch but no inhibition, the input weight of stretch was increased from 0.25 to 0.7 and the model was evaluated at t = 24 h. For the unstretched and stretched inhibited conditions, y_i_^max^ corresponding to the AT_1_R node was set to 0 before applying changes to the stretch input weight and was evaluated at t = 24 h. The same combinations were used for the other four set of simulations on 3 kPa substrate conditions, but with a stiffness input weight of 0.7.

The implementation of the model in Python 3.9.0 along with simulation data are available on Github.

### 2.8. Statistics

Descriptive statistics were performed using JMP Pro Statistical software (version 14, SAS Institute Inc., NC, USA) for group comparisons of relative gene expression. For normally distributed data, one-way analysis of variance (ANOVA) was used to test for differences in means of three different stiffnesses and gene expression of the normotensive pulmonary artery adventitial layer for all six genes, followed by the Dunnett’s post-hoc test. Otherwise, the non-parametric Wilcoxon–Kruskal–Wallis statistic was used followed by Dunnett’s post-hoc test. Effects of stiffness and stretch were tested using two-way ANOVA with stiffness and stretch as fixed factors. For normally distributed data, the Dunnett’s post-hoc test was used. Otherwise, the non-parametric Wilcoxon–Kruskal–Wallis statistic was used followed by Dunnett’s post-hoc test. For the inhibition studies, three-way ANOVA was used to compare the effects of stiffness, stretch, and inhibition, followed by Sidak’s post-hoc test. Data are expressed as means ± standard error of the mean, unless otherwise specified. Statistical significance was determined at a level of 0.05. Data were graphed in GraphPad Prism software (v8.4.3.686, San Diego, CA, USA) and Illustrator (Adobe, San Diego, CA, USA, v24.2.3).

## 3. Results

### 3.1. PAAFs Upregulate Profibrotic Genes in Response to Increased Substrate Stiffness and Stretch

**Figure 2:**
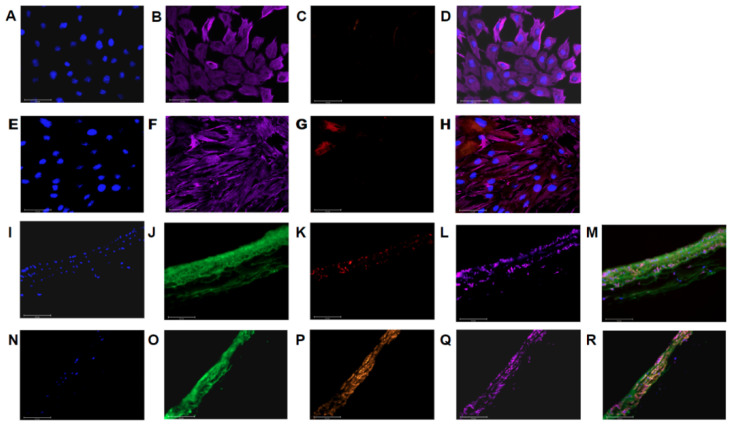
The cell culture showed positive labeling for (**A**,**E**) DAPI, (**B**,**F**) vimentin, (**C**) vWF, and (**G**) MYH11 with (**D**,**H**) overlays. Out of 425 isolated cells, 3% expressed vWF, and out of 888 cells, 0.2% expressed MYH11 and 100% expressed vimentin with representative images shown in (**A**–**H**). Immunostained PA tissue sections showed positive labeling for: (**I**) DAPI, (**J**) WGA, (**K**) vWF, (red), and (**L**) vimentin(magenta) with an (**M**) overlay. Separate immunostained PA tissue sections showed positive labeling for: (**N**) DAPI, (**O**) WGA, (**P**) MYH11 (orange), and (**Q**) vimentin (magenta) with an (**R**) overlay. These samples were used as labeling controls to estimate purity of a cell culture expanded on plastic. Images were all acquired at 40× magnification, scale bar 50 µm.

When we immunostained cultures for markers of endothelial cells (vWF), smooth muscle cells (MYH11), and myofibroblasts (vimentin) (Figure 2A–H), only 3% were positive for vWF and 0.2% were positive for MYH11 suggesting high enrichment of PAAFs in our cell cultures. Intact pulmonary artery tissue sections were stained and imaged with the same antibodies and imaging settings as positive controls for these markers (Figure 2I–R).

**Figure 3:**
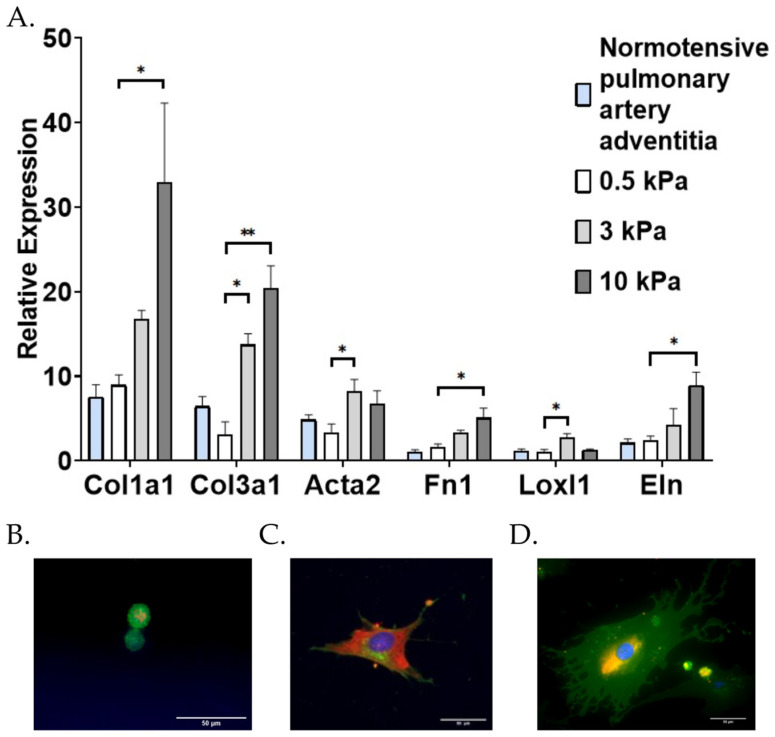
Effect of substrate stiffness on pulmonary arterial adventitial fibroblast (PAAF) differentiation. (**A**) Mean ± standard errors of the mean relative to housekeeping gene 18S ribosomal RNA of PAAFs cultured at different stiffness (*n* = 9) compared with gene expression of sections in a normotensive pulmonary artery adventitia (*n* = 6). Effect of stiffness (* *p* < 0.05 and ** *p* < 0.0001) by one-way analysis of variance (ANOVA) compared with control 0.5 kPa with a post-hoc Dunnett’s test. (**B**–**D**) PAAFs plated on (**B**) 0.5 kPa (40×), (**C**) 3 kPa (20×) polyacrylamide gel and (**D**) plastic (40×), scale bar 50 µm. Cells were stained with DAPI, which stains the nucleus (blue), wheat germ agglutinin stains the membrane (green), andα-SMA filaments (orange).

**Figure A1:**
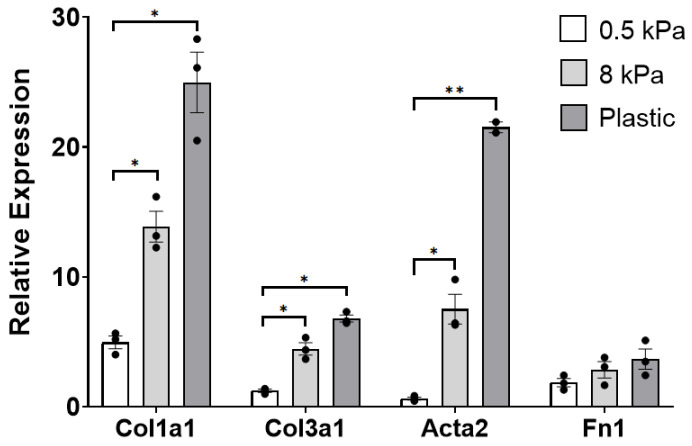
Mean ± standard errors of the mean of *Col1a1*, *Col3a1*, *Acta2*, and *Fn1* gene expression of cells seeded on Cytosoft^®^ plates. Effects of stiffness on PAAF gene expression relative to 18S ribosomal RNA. Significant effect of stiffness (* *p* < 0.05 and ** *p* < 0.0001) by one-way analysis of variance (ANOVA) compared with gene expression at 0.5 kPa.

PAAFs expanded on plastic reverted from a myofibroblast to a fibroblast phenotype after three days of culture on 0.5 kPa stiffness 6-well plates, as assessed by their low expression of *Acta2* (Appendix A, Figure A1) and their rounded appearance in culture (Figure 3B) compared with the more stellate shapes and higher *Acta2* expression in cells grown on stiffer substrates (Figure 3C,D). Messenger RNA levels of *Col1a1*, *Col3a1*, *Eln*, *Fn1*, *Loxl1*, and *Acta2* genes in fibroblasts cultured on 0.5 kPa substrates were not significantly different from those obtained by extracting RNA from the pulmonary artery adventitia of a normotensive rat (*p* > 0.05 by one-way ANOVA, Figure 3A). This finding suggests that cells cultured on a 0.5 kPa substrate may mimic expression of PAAFs in vivo with respect to the six genes studied in this paper.

Compared with mRNA levels in PAAFs cultured on 0.5 kPa substrates, all six genes were significantly upregulated in response to increased matrix stiffness (*p* < 0.05 by one-way ANOVA and Dunnett’s post-hoc test). The expression of *Acta2* and *Loxl1* was significantly higher on cells grown on 3 kPa matrices but not significantly higher on cells grown on 10 kPa matrices, while *Col1a1*, *Col3a1*, *Eln*, and *Fn1* were significantly upregulated on 10 kPa substrates (comparable to arterial stiffness in advanced PAH [14]), compared with PAAFs cultured on 0.5 kPa matrices (Figure 3A). Interestingly, *Acta2* and *Loxl1* expression exhibited non-monotonic responses, with significant upregulation of gene expression on 3 kPa matrices compared with the 0.5 kPa matrices, but no significant difference between cells cultured on 0.5 kPa and 10 kPa matrices.

**Figure 4:**
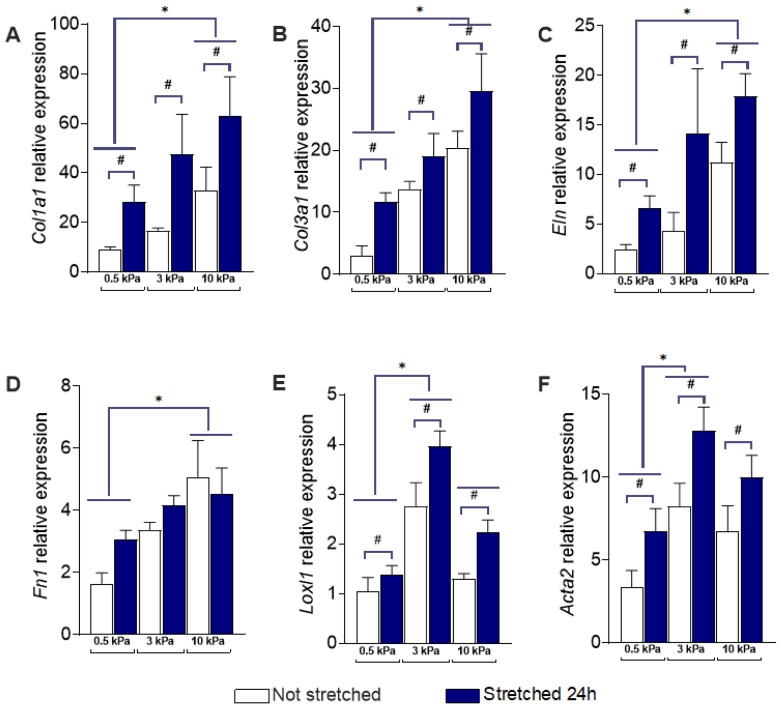
Effect of stiffness and stretch on gene expression in pulmonary arterial adventitial fibroblasts (PAAFs). Mean ± standard errors of the mean of mRNA levels relative to housekeeping control gene 18S ribosomal RNA in unstretched cells (*n* = 9, white bars) and after 24 h 10% equibiaxial stretch (*n* = 12, blue bars). * Significant pairwise effect of stiffness (*p* < 0.05) by a post-hoc Dunnett’s multiple comparisons test and # significant effect of stretch (*p* < 0.05) based on group comparisons made using a two-way analysis of variance (ANOVA) for: (**A**) Collagen I (*Col1a1*) (**B**) Collagen III (*Col3a1*) (**C**) Elastin (*Eln*) (**D**) Fibronectin (*Fn1*) (**E**) Lysyl oxidase-like 1 (*Loxl1*) (**F**) Smooth Muscle Actin (*Acta2*).

Examining the transcriptional responses of the six genes to 10% equibiaxial stretch for 24 h in PAAFs (Figure 4A–F), *Col1a1*, *Col3a1*, *Eln*, *Loxl1,* and *Acta2* were significantly upregulated compared with unstretched cells independent of the substrate stiffness (*p* < 0.05 based on group comparisons made using a two-way ANOVA). Although *Fn1* expression did not significantly change after 24 h of stretch (Figure 3D), it was significantly upregulated after 4 h on all gel stiffnesses (from 1.62 ± 0.34 to 5.07 ± 1.22 on 0.5 kPa gels, 3.36 ± 0.26 to 6.93 ± 1.65 on 3 kPa gels and 5.08 ± 1.16 to 8.04 ± 2.32 on 10 kPa gels, *p* = 0.0002, *n* = 6). On the other hand, *Col1a1* was only significantly upregulated after 24 h of stretch, but not after 4 h. This suggests *Fn1* is transiently induced by a short period of stretch, while the *Col1a1* response to stretch is much slower. This finding is consistent with reports identifying *Fn1* as an early response gene [40]. No significant interaction effects between substrate stiffness and stretch were found in the expression of any of the six genes (Table 1).

**Figure 5:**
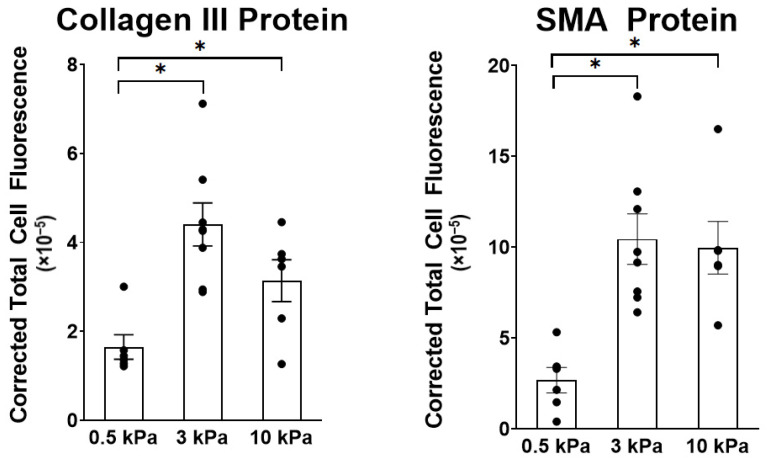
Effect of stiffness on protein expression of Collagen III and Smooth Muscle Actin (SMA) in PAAFs. Mean ± standard errors of the mean of Corrected Total Cell Fluorescence (CTCF) for 6–8 replicate hydrogels. * Significant pairwise effect of stiffness (*p* < 0.05) determined by one-way analysis of variance (ANOVA).

Increased ECM stiffness significantly upregulated protein expression of Collagen III and Smooth Muscle Actin (SMA) from a baseline of 0.5 kPa at both 3 kPa and 10 kPa based on a post-hoc test (Figure 5). There was no significant difference between 3 kPa and 10 kPa protein expression for either Collagen III or SMA. This is consistent with the relative expression of RNA for Collagen III (*Col3a1*) and SMA (*Acta2*), shown in Figure 4B,F, which significantly increased from 0.5 kPa to 3 kPa, without further significant increase from 3 kPa and 10 kPa substrates.

### 3.2. PAAF Network Model Simulates Gene Expression Activated by Stiffness and Stretch

**Figure 6:**
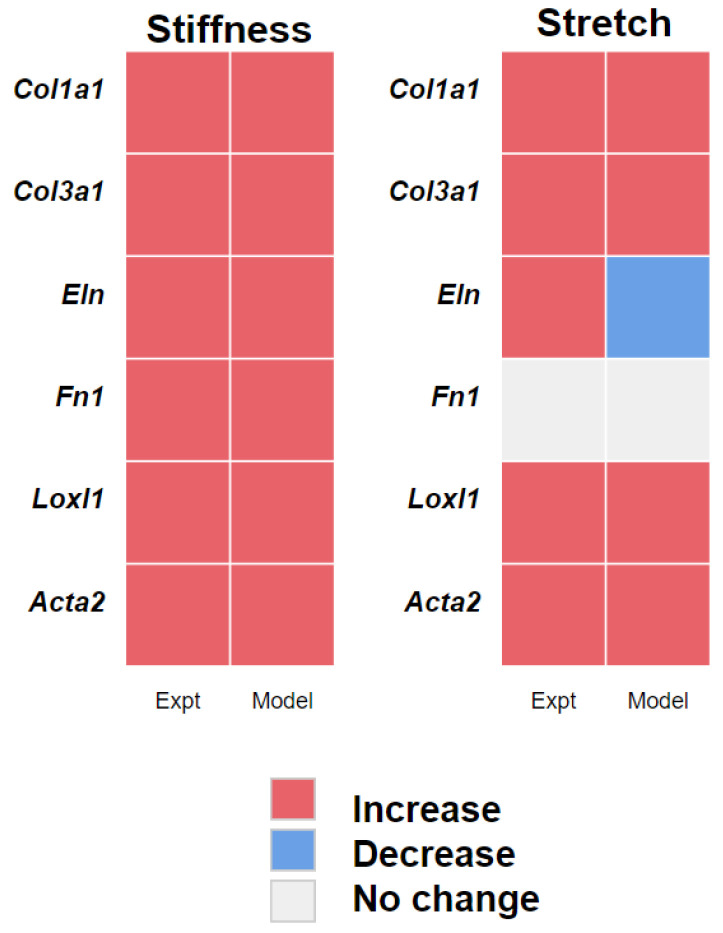
Comparison of the experimental observations (Expt) with model predictions (Model) of gene activity due to stretch and stiffness. Increase (red), decrease (blue), and no change (grey) in gene expression predicted by the model is based on a threshold of 0.1 and experimental observations that reached a significant difference 24 h after stretch (*p* < 0.05).

A threshold change of 0.1 in the normalized variable representing each of the six genes was used to classify the change in each gene as significant. The model predicted significant upregulation of all six genes in response to an increase in substrate stiffness from 0.5 kPa to 3- or 10 kPa (Figure 6). These model predictions matched our experimental observation that all six genes were significantly upregulated in cells grown on stiffer matrices.

The model also predicted upregulation of the gene expression of *Col1a1*, *Col3a1*, *Loxl1*, and *Acta2* and the return to baseline of *Fn1* expression 24 h after induction by 10% equibiaxial stretch. However, the model predicted *Eln* expression to be downregulated with stretch while experimental results showed upregulation. We investigated whether the inhibitory effect of JNK1/2 on *Eln* may have outweighed the activating effect of PKC and found that decreasing the weight of the inhibition of JNK1/2 on *Eln* [41] by 50% allowed the model to predict the observed upregulation of *Eln* (Figure 4). While the model was in qualitative agreement with the data, it did not recapitulate the non-monotonic responses of *Loxl1* and *Acta2* (Figure 3A), which were significantly upregulated by 3 kPa matrix stiffness (compared with 0.5 kPa) but not by 10 kPa substrates.

### 3.3. Angiotensin II Receptor Inhibition Unmasks an Interaction Between Stiffness and Stretch on Fibronectin Gene Expression

Based on a sensitivity analysis [12], we simulated the effects of stretch and increased stiffness in the presence of inhibitors of three mechanosensitive nodes in the model (AT_1_, and TGF-β, and MST1/2). Table 2 shows the effects of inhibiting AT_1_, TGF-β, and MST1/2 on changes in gene expression due to an increase in substrate stiffness from 0.5 and 3 kPa and due to stretch on 0.5 kPa stiffness matrices. Here, model-predicted differences in the normalized mRNA variable due to inhibitor treatments were considered significant if they exceeded a threshold of 0.1.

From the model simulations, the induction of *Loxl1* expression by increased substrate stiffness is specifically regulated by MST1/2 signaling, whereas the responses of the other five genes to stiffness were all significantly inhibited by blocking TGF-β receptor. AT_1_ receptor inhibition significantly attenuated the stiffness-dependent induction of *Col1a1*, *Col3a1*, *Fn1,* and *Acta2*, but had no significant effect on *Eln* or *Loxl1*, and the magnitude of inhibition was noticeably less than when TGF-β receptors were blocked. Blocking angiotensin signaling in the model with increased substrate stiffness downregulated the collagens by 20% and blocking TGF-β signaling downregulated the collagens by 30%, while blocking angiotensin downregulated *Acta2* by 17% and blocking TGF-β downregulated it by 86% (Table 2).

Blocking TGF-β signaling in the model while applying stretch stimulation suppressed the upregulation of *Acta2* by 28% and reduced the downregulation of *Eln* by 11%. Stretch induction of *Col1a1* and *Col3a1* was shown to be reduced by inhibition of MST1/2 (by 46%) and angiotensin II signaling (by 26%), while *Loxl1* regulation by stretch was affected only by inhibiting MST1/2 (by 100%). *Fn1* expression, which was not significantly altered by stretch, remained unchanged in the presence of all three inhibitors. This is in contrast to its response to substrate stiffness, where inhibiting AT_1_ and TGF-β receptors had a significant effect (Table 2).

**Figure 7:**
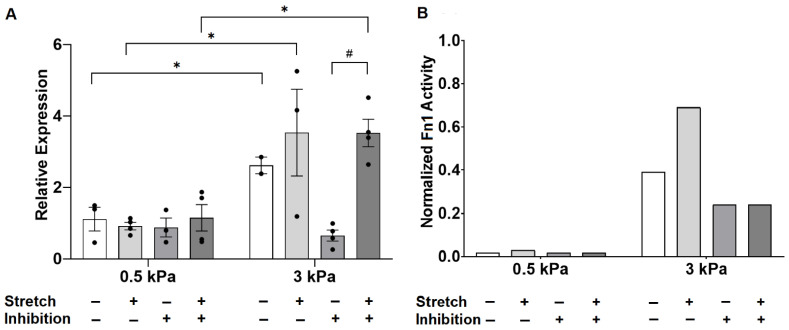
Experimental observations of fibronectin gene expression in response to increased substrate stiffness and 10% equibiaxial stretch, with and without AT_1_ receptor inhibitor losartan. (**A**) Data are expressed as *Fn1* gene expression mean ± standard errors relative to 18S ribosomal RNA housekeeping gene. * Significant effect of stiffness (*p* < 0.05), # significant effect of stretch (*p* < 0.05) by three-way analysis of variance (ANOVA) with a post-hoc Sidak’s test. (**B**) Model simulation results where stiffness and stretch stimuli were increased by increasing the stiffness input weight from 0.25 to 0.7, stretch input weight from 0.25 to 0.7, and inhibition applied by blocking the AT_1_R node.

Since the model suggested a significant role for angiotensin II signaling in regulating *Fn1* expression in response to increased stiffness but not stretch (Table 2 and Figure 7B), we treated cultured PAAFs with 1 M losartan, an AT_1_ receptor blocker.

Losartan abrogated the induction of fibronectin mRNA expression by 3 kPa substrates compared with 0.5 kPa, and *Fn1* expression by PAAFs grown on 0.5 kPa matrices remained unresponsive to stretch after 24 h (Figure 7A). Stretch significantly upregulated the expression of *Fn1* by PAAFs grown on stiffer 3 kPa substrates when angiotensin II signaling was blocked.

Model simulations of *Fn1* expression in response to increased stiffness, stretch, and AT_1_ receptor inhibition corresponding to each experimental condition in Figure 7A are shown in Figure 7B. The model recapitulated the increase in fibronectin mRNA under control conditions, when substrate stiffness increased from 0.5 kPa to 3 kPa, and the qualitative response of *Fn1* on 3 kPa matrices to induction by stretch. The model also correctly predicted no effect of losartan on *Fn1* mRNA levels under stretched and unstretched conditions on 0.5 kPa substrates 24 h after stretch. However, while we observed in vitro that losartan inhibited *Fn1* upregulation by increased stiffness in unstretched but not stretched PAAFs, the model could not reproduce this observation.

## 4. Discussion

In-vitro experiments in pulmonary arterial adventitial fibroblasts (PAAFs) were used to investigate the differential effects of equibiaxial stretch and increased substrate stiffness on six genes of a new mathematical model of PAAF cell signaling [12]. While both physical stimuli occur in PAH, these stimuli are thought to occur at different stages of the disease, in part because increased vascular fibrosis leads to ECM stiffening that in turn opposes the increase in arterial wall strain caused by increased wall stress. In this study, we used a novel combination of in-vitro and in-silico models to investigate how PAAFs respond to changes in ECM stiffness and strains representative of those associated with adventitial remodeling in PAH. While PAAFs are exposed to cyclic loading in vivo, we used static stretch as a model of the chronically elevated mean hemodynamic load (Herum KM et al., 2017) during PAH rather than acute phasic vascular loading, in part because cyclic stretch systems cannot recapitulate the high physiological cardiac frequencies (6 Hz) in rats (Layland J et al., 1995) [10,42]. Although there are no existing studies examining how PAAFs respond to cyclic stretch, Wu et al. (Wu J et al., 2013) reported that 10% cyclic stretch for 36 h led to 2–3 fold increases in *Col1a1* and *Col3a1* expression in mouse aortic fibroblasts, which are comparable to the 3-fold increase in *Col1a1* and 2- to 4-fold increase in *Col3a1* that we observed after 24 h of static stretch [43]. While the cells were maintained at a 10% static stretch for 24 h, measurements of cell area in cardiac fibroblasts using the same circular custom stretchers (Herum KM et al., 2017) showed that after cell area initially increased during static stretch, they returned to their original size within 1 h, well before the 24-h time point at which gene expression was measured [10].

### 4.1. Stiffness and Stretch Differentially Affect Expression of Six Profibrotic Genes

Stretch and increased substrate stiffness were both able to upregulate five out of the six profibrotic genes we investigated. However, while increasing stiffness from 0.5 kPa significantly induced all six genes, fibronectin expression was transiently upregulated by stretch at 4 h but was not significantly altered by stretch at 24 h. There was also a non-monotonic response to the two levels of increased substrate stiffness in the expression of *Loxl1* and *Acta2*, which were both upregulated compared with 0.5 kPa substrates on 3 kPa matrices (similar to vessel walls during mild PAH), but the expression of both was not significantly altered compared with 0.5 kPa substrates on 10 kPa matrices (which are comparable in stiffness to vessel walls during severe PAH). Unlike observations in cardiac fibroblasts [10], we found no statistical interaction effects between the stretch and stiffness conditions in these six genes. These results suggest that the expression of *Col1a1*, *Col3a1*, and *Eln* could be expected to rise early in vivo as elevated pulmonary arterial pressure increases vascular wall strain and remain elevated as fibrosis increases adventitial ECM stiffness, even though this stiffening would also reduce arterial strain. In contrast, *Loxl1* and *Acta2* expression may initially rise but eventually return to baseline as wall stiffening becomes severe, and *Fn1* mRNA may be induced only after the ECM has remodeled and stiffened.

### 4.2. Model Modifications to Investigate Differential Regulation by Stretch and Stiffness

By allowing stiffness and stretch to be separate inputs to the model, we investigated the pathways regulating the expression of six mechanosensitive genes (*Col1a1*, *Col3a1*, *Eln*, *Fn1*, *Loxl1*, *Acta2*) in response to each stimulus. While there is published evidence that TGF-β is activated by stretch in cardiac [10] and lung [44] fibroblasts, we only found experimental evidence of TGF-β activation by substrate stiffness in PAAFs [23,24]. Based on ample published data in other cell types, we refined the model of the MAPK signaling cascade in the original version of our model so that ERK1/2, p38, and JNK1/2 could be independently activated, and we updated the model to include the effects of stretch activated TRP channels observed by Yue and Suzuma et al. [37,38].

Comparing the predictions of this revised model against the same independent experimental data from rat and human PAAFs that we used to test our original implementation [12], we found no significant changes in model validation accuracy from what we reported previously [12]. Comparing predictions of the revised model with in-vitro PAAF experiments conducted here on the effects of stretch and stiffness on gene expression, the model correctly predicted the upregulation of all six ECM genes by increased stiffness though not the subsequent return to baseline levels on the stiffest matrices for *Loxl1* and Acta2. The model also correctly predicted the observed upregulation of four ECM genes and the lack of response to stretch in *Fn1* expression at 24 hrs. However, while we observed an increase in *Eln* mRNA after stretch, the model incorrectly predicted a decrease. Examining the regulation of elastin gene expression in the network, we found that halving the weight of JNK1/2 inhibition on *Eln* mRNA while leaving the activating weight of PKC on *Eln* the same reversed this result. Hence it is possible that the activating effect of PKC dominates the inhibiting effect of JNK1/2 in the regulation of elastin gene expression by stretch.

The equations in the model were formulated using studies from both in-vivo and in-vitro experiments. While we used the rat PAAFs to validate the gene expression in response to stimuli such as mechanical stretch or substrate stiffness, this approach allows us to predict how phenotypic outputs respond to mechanical load. It is reported that mechanical stretch may increase the stiffness of the substrate, which in turn decreases the stretch. However, the interactions between them have not yet been classified. Through this work, we can model the interactions by adjusting time parameters and the activated reactions to represent beneficial versus maladaptive remodeling in fibrosis. Furthermore, the model can simulate a high number of experimental designs and make corresponding predictions that would be difficult to reproduce experimentally. This feature also enables the model to predict effects of specific drugs through simulating the activation or inhibition of any target species in the network.

### 4.3. Crosstalk between TGF-β and Angiotensin II

Using this model to predict the effects of inhibiting key mechanoresponsive nodes in the network, we found that blocking AT_1_R in the model significantly decreased expression of *Fn1* in response to stiffness but did not significantly decrease expression of *Fn1* in response to stretch. Experimentally, we confirmed that blocking the AT_1_ receptor with losartan inhibited the significant upregulation of *Fn1* expression when substrate stiffness is increased and had no effect on the response to stretch on 0.5 kPa substrates. However, losartan unmasked a response to stretch in PAAFs grown on the stiffer matrices that was not seen in untreated cells or predicted by the model. These findings show that angiotensin II signaling is required for the *Fn1* response to increased stiffness, and that *Fn1* expression can be stretch-regulated by an angiotensin-independent pathway on stiffer matrices when the saturating effects of higher stiffness are blocked. The requirement for angiotensin receptors to be activated before fibronectin mRNA can be induced by elevated substrate stiffness may be related to angiotensin-mediated conversion of latent TGF-β to the active state [45,46]. The network already represents this feedback based on reports that cyclic strain activates AT_1_R to cause activation of TGF-β in rat cardiac fibroblasts and human fibroblasts [45,47]. However, because stiffness also directly activates TGF-β in the model, angiotensin signaling could be blocked in the model without preventing stiffness from inducing *Fn1* expression. This suggests that increased stiffness only activates TGF-β signaling after angiotensin has activated latent TGF-β in our experiments. Angiotensin receptor inhibition also unmasked angiotensin-independent *Fn1* expression in response to stretch at a higher substrate stiffness, but in the model, fibronectin mRNA can only be induced by stretch directly via the angiotensin receptor. Hence, our experimental results suggest that there is another stretch-activated pathway that regulates a transient response to stretch in fibronectin and is more active in cells grown on stiffer matrices independent of angiotensin II. This indicates a currently unknown angiotensin-independent stretch-activated pathway responsible for an initial rapid upregulation of fibronectin gene expression, possibly the STAT3 pathway, which is involved in stretch induction of fibronectin in renal epithelial cells though not in fibroblasts [48]. An angiotensin-dependent pathway that downregulates fibronectin expression after 4 h should also be added to the model. One way to further examine this potential crosstalk would be to treat PAAFs with a TGF-β blocker based on the high magnitude of inhibition predicted by the model simulations (Table 2). When blocking TGF-β in the model, expression of *Col1a1*, *Col3a1*, *Eln*, *Fn1*, and *Acta2* was significantly inhibited in response to increased stiffness, with no significant changes observed on inhibiting stretch effects except for *Acta2*.

### 4.4. Limitations

We used rat PAAFs because of the detailed biomechanical measurements of ECM stiffness pulmonary arterial strain in the sugen-hypoxia rat model of PAH and normotensive control rats. However, human PAAF cell lines have been used to study fibrotic signaling in response to increased ECM stiffness [11], where they showed that ten out of twelve genes studied were differentially expressed when stiffness increased from 1 to 12 kPa. Their analysis identified an miR-130/301-PPARγ signaling network regulated by ECM stiffness and associated with ECM remodeling in human PAH. Studies of mechanosignaling in human PAAF cell lines would enable us to generate a similar model of profibrotic mechanosignaling in human cells that could include these networks. ECM remodeling depends on protein synthesis, post-translational modifications, and cell-mediated matrix assembly [2]. One limitation of this study is that we focused primarily on gene expression, but we did find that changes in collagen III and smooth muscle actin protein abundances in response to increased ECM stiffness were consistent with changes in their mRNA expression. Finally, while the model was able to predict the response of fibronectin gene expression on soft gels, it did not replicate all of the observed responses to AT_1_ receptor inhibition on stiff gels or the transient response of *Fn1* expression to stretch. These model limitations can nevertheless be used to identify candidate pathways and reactions that need to be added to the network.

## 5. Conclusions

In-vitro experiments using hydrogel substrates of various stiffnesses coating elastic membranes in cell-stretch devices showed that expression of profibrotic genes by PAAFs is differentially regulated by cell stretch and extracellular matrix stiffness. No interaction effects between stretch and stiffness were observed for the six genes studied here; however, the AT_1_ receptor blockade uncovered an angiotensin-independent activation of *Fn1* expression by stretch in PAAFs when grown on stiff but not soft substrates. A novel combination of in vitro and in silico models of PAAF profibrotic cell signaling in response to altered mechanical conditions may help identify regulators of the vascular adventitial remodeling that results from the changes in stretch and matrix stiffness occurring during the progression of PAH in vivo.

## Figures and Tables

**Table 1:** Calculated *p*-values from two-way analysis of variance (ANOVA) of the effects of substrate stiffness and stretch on the expression of six genes in cultured pulmonary arterial adventitial fibroblasts (PAAFs). Bolded values indicate *p* < 0.05.

Genes	Effects of Stiffness	Effect of Stretch	Interaction Term
*Col1a1*	**0.046**	**0.006**	0.86
*Col3a1*	**<0.0001**	**0.012**	0.69
*Eln*	**0.009**	**0.009**	0.66
*Fn1*	**0.001**	0.27	0.28
*Loxl1*	**<0.0001**	**0.0007**	0.30
*Acta2*	**0.0007**	**0.002**	0.88

**Table 2:** Changes in gene expression due to inhibition of Angiotensin II Receptor Type I (AT_1_), Transforming Growth Factor-β (TGF-β), and Macrophage Stimulating 1 or 2 (MST1/2) receptors in response to stiffness and stretch. Numbers in bold indicate activity changes greater than a threshold of 0.1.

	Effect of Stiffness on 3 kPa			Effect of Stretch on 0.5 kPa		
Genes	AT1	TGF-β	MST1/2	AT1	TGF-β	MST1/2
*Col1a1*	**−0.16**	**−0.24**	**−0.10**	**−0.14**	−0.06	**−0.26**
*Col3a1*	**−0.16**	**−0.24**	**−0.10**	**−0.14**	−0.06	**−0.26**
*Eln*	−0.04	**−0.46**	0	**0.20**	−0.08	0
*Fn1*	**−0.15**	**−0.39**	0	−0.01	−0.03	0
*Loxl1*	0	0	**−0.37**	0	0	**−0.37**
*Acta2*	**−0.14**	**−0.70**	0	−0.04	**−0.14**	0

## Data Availability

Data can be found in the supplementary files section: at https://www.mdpi.com/article/10.3390/cells10051000/s1 (accessed on 21 April 2021) and at https://github.com/dvaldezjasso/DVJ-LAB/tree/main/PAAF (accessed on 21 April 2021).

## Supplementary Materials

The following are available online at https://www.mdpi.com/article/10.3390/cells10051000/s1, Table S1: Primers used to run RT-PCR are listed, as are protocols for conversion of RNA to cDNA, the PCR setup, raw data from the StepOnePlus^TM^ Real-time PCR machine, and analyzed data for all RT-PCR experiments in this paper, DVJ 2021 PAAF stiffness Gel Stretch RT-PCR.xlsx; Table S2: Imaging data for Collagen III and SMA, IntDenCTCFActa2Col3.xlsx.

## Abbreviations

PAH	Pulmonary Arterial Hypertension
PAAF	Pulmonary Arterial Adventitial Fibroblast
TGF-β	Transforming Growth Factor Beta
ECM	Extracellular Matrix
Ang II	Angiotensin II
AT_1_R	Angiotension II Receptor Type 1
MST1/2	Macrophage Stimulating 1 or 2
TRP	Transient Receptor Potential Ion Channel
Col	Collagen mRNA
Acta2	Alpha Smooth Muscle Actin mRNA
Loxl1	Lysyl oxidase-like 1 mRNA
Eln	Elastin mRNA
Fn1	Fibronectin mRNA
ANOVA	Analysis of variance
JNK1/2	c-Jun N-terminal Kinases
PKCα	Protein Kinase C Alpha
ROS	Reactive Oxygen Species
ASK1	Apoptosis Signal-regulating Kinase 1
TAK1	TGF-β–Activated Kinase 1
